# Daptomycin and Its Immunomodulatory Effect: Consequences for Antibiotic Treatment of Methicillin-Resistant *Staphylococcus aureus* Wound Infections after Heart Surgery

**DOI:** 10.3389/fimmu.2014.00097

**Published:** 2014-03-11

**Authors:** Theodor Tirilomis

**Affiliations:** ^1^Department of Thoracic, Cardiac, and Vascular Surgery, University of Göttingen, Göttingen, Germany

**Keywords:** cardiac surgery, antibiotic treatment, wound debridement, resistant bacteria, *Staphylococcus aureus*, mediastinitis

## Abstract

Infections by methicillin-resistant *Staphylococcus aureus* (MRSA) play an increasing role in the postoperative course. Although wound infections after cardiac surgery are rare, the outcome is limited by the prolonged treatment with high mortality. Not only surgical debridement is crucial, but also antibiotic support. Next to vancomycin and linezolid, daptomycin gains increasing importance. Although clinical evidence is limited, daptomycin has immunomodulatory properties, resulting in the suppression of cytokine expression after host immune response stimulation by MRSA. Experimental studies showed an improved efficacy of daptomycin in combination with administration of vitamin E before infecting wounds by MRSA.

Infections with methicillin-resistant *Staphylococcus aureus* (MRSA) are an increasing problem worldwide and although MRSA is less prevalent in northern Europe, its appearance in hospitals and especially in intensive care unit is highly problematic (1, 2). MRSA infections after cardiac surgery are responsible for high in-hospital mortality (3). In general, infections after heart surgery may be systemic infections but also quite often related to the wounds (Figure 1). Postoperative pneumonia is mainly ventilator associated and the risk is increased with prolonged duration of postoperative mechanical ventilation (4, 5). Bloodstream infections after open-heart surgeries are very rare (6, 7). These infections are probably related to transfusion and especially to red blood cell units that are older than 14 days (6). Contamination of the cardiopulmonary circuit and its compounds seems to be less important. Although in the study of Hamers and colleagues positive cultures from cardiopulmonary bypasses were not a rarity, they did not register increased risk of postoperative infections (8). The data regarding postoperative endocarditis are comparable to bacteriemia; sporadic cases have been reported. Wound infections after open-heart surgery are of the most serious complications. Deep wound infections are post-sternotomy associated and may involve the mediastinal area and the sternal bone (Figure 2). The incidence of postoperative mediastinal wound infection is mostly around 2% but may rise up to 5% (9–13). The “mechanisms” of wound infection are (i) peri-operative contamination, (ii) spread from concomitant infection, or (iii) associated with comorbidities (e.g., chronic obstructive lung disease). *S. aureus* mediastinal infections are mainly caused by peri-operative contamination (12). Due to increased postoperative mortality of around 20%, deep wound infections and mediastinitis remain a very serious complication after cardiac surgery (12).

**Figure 1:**
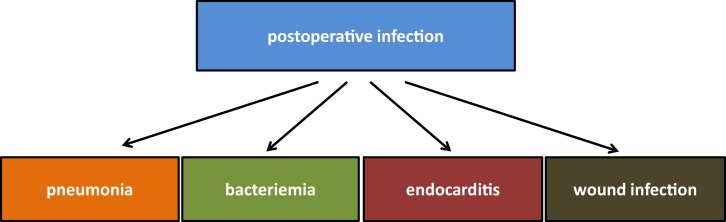
**Possible infections after cardiac surgery**.

**Figure 2:**
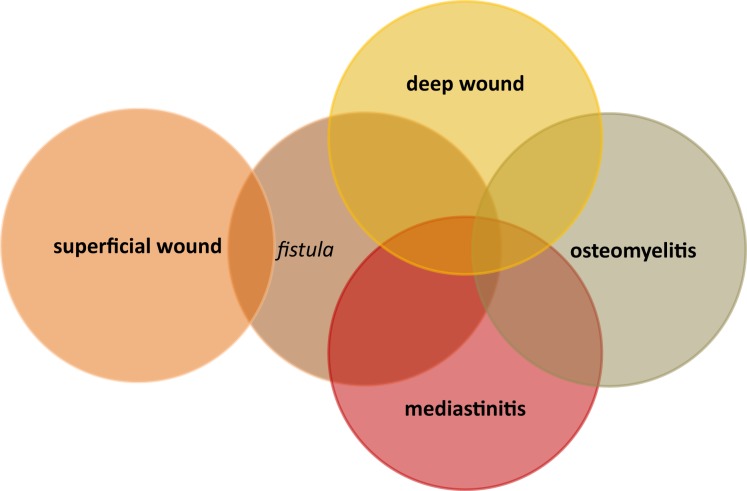
**Types and interactions of post-sternotomy wound infections**.

In case of MRSA-related post-sternotomy infection, even long-term survival was significantly reduced (14, 15). In contrast to these findings, in a recently published study of 4,722 patients who underwent coronary artery bypass grafting, Colombier et al. did not find that long-term survival was influenced by MRSA infections (15). Nevertheless, they reported in-hospital mortality three times higher than in patients without deep wound infections (16). However, early and aggressive treatment is crucial. Despite preventing strategies and surgical treatment, including removal of foreign material and extended wound debridement, antibiotics are an essential part of the therapy. In case of MRSA, antimicrobial therapy is limited due to the resistance to many antibiotics, including third-generation-cephalosporins, macrolides, and quinolones. The intravenous administration of vancomycin is the primary therapeutic option for MRSA infections (17). Vancomycin is a glycopeptide inhibiting the synthesis of the bacterial cell wall. Carrier et al. reported the implementation of many strategies to control contamination after an outbreak of MRSA mediastinal infections (18). They reduced the incidence of MRSA infection and mediastinitis after cardiac surgery among patients with MRSA infection or nasal carriers of MRSA, giving prophylactic vancomycin along with preventing isolation and nasal application of mupirocin. In a decision analysis model to estimate surgical site infections in cardiothoracic surgery, Miller and colleagues found the risk of surgical site MRSA infections after prophylactic application of vancomycin and therefore they suggest routine prophylaxis with vancomycin (19). Walsh et al. gave in their MRSA intervention program vancomycin prophylaxis for identified MRSA carriers along with intranasal mupirocin application to all patients regardless of colonization status (20). Their strategy resulted in a near-complete elimination of MRSA wound infections after cardiac surgery. Unselected, general prophylaxis with vancomycin may be problematic. In this context, Bull et al. found in a large analysis of 22,549 procedures, including aortocoronary bypass grafting and orthopedic procedures, an increased risk of wound infections caused by methicillin-sensitive *S. aureus* strains (21). However, also development of intermediate glycopeptide-resistant strains has been reported (22). Linezolid is an alternative to vancomycin and is, in contrast to vancomycin, completely bioactive after oral administration. The oral application is associated with increased patient comfort especially in patients with long-lasting treatment and in cases treated in the out-patient department. The efficacy of both treatments, vancomycin and linezolid, is comparable (23, 24). Although both agents were well tolerated, thrombocytopenia, anemia, diarrhea, and nausea were more often presented in patients treated by linezolid (25). The biggest problem in soft-tissue infections is the tissue penetration of the antimicrobial drugs. Distribution of vancomycin into soft tissue is variable (26). Concentrations of linezolid in soft tissue are similar or higher than plasma levels, although some variability exists.

Daptomycin, a novel cyclic lipopeptide antibiotic, is effective in soft-tissue infections, in hospitalized patients, and in community-acquired infections (27). Penetration of daptomycin into soft tissue is good, with reported concentrations of more than 70% of the plasma level within 2 h of administration and maintenance of these levels for 12 h (28). Early experimental studies demonstrated efficacy of daptomycin also in a pneumonia model, but the effect was no superior to vancomycin treatment (29). Because daptomycin had failed to meet the statistical endpoint in a phase 3 trial for the treatment of community-acquired pneumonia, this antimicrobial is not indicated for use in the treatment of pneumonia (30, 31). Daptomycin has shown to be effective even for treatment of MRSA wound infections in cardiac surgery (32). The basic characteristics of vancomycin, linezolid, and daptomycin are summarized in Table 1. The overall clinical success rates of daptomycin therapy were reported above 90% (33). Diabetes and severe renal impairment along with andocarditis and bacteriemia were associated with higher rates of clinical failure (33). Also regarding surgical wound infections caused by MRSA, the success rates were comparable high, regardless of the depth of the infection (34). In wound infections, the daily dosage of daptomycin is usually 4 mg/min. However, in multicenter, retrospective studies high-dose daptomycin (daily more than 8 mg/kg) were effective in complicating infections, including wound infections, with low adverse events (35, 36). Daptomycin has bactericidal activity caused by the calcium-dependent release of potassium and membrane potential dissipation. This mechanism is ultimately leading to cytoplasmic membrane disruption and cell death (37). Pogliano and colleagues found that the membrane defects occur rapidly within 15 min (38). They observed also that daptomycin inserts preferentially in the leading edges of the septal and forespore membranes, also inducing changes in the membrane structure. Furthermore, they found relocalization of the peptidoglycan biogenesis apparatus. Along with the reorganization of the membranes, daptomycin is responsible for the cell shape and membrane alterations. Therefore, daptomycin is likely to be directly responsible for mislocalization of essential cell division proteins (38). Recently, Berti and colleagues found enhanced activity of daptomycin against MRSA in the presence of β-lactam antibiotics (39). Although the exact mechanism is not clear, this effect was related to the interference of the β-lactam antibiotics (in subinhibitory concentrations) with the penicillin-binding protein 1. Furthermore, daptomycin was more effective in inhibiting MRSA in biofilm than linezolid and vancomycin (40). After surgery and especially after cardiac surgery with implants and osteosynthetic material, biofilm formation by antibiotics in subminimal inhibitory concentrations may play a significant role (41). Recently, the efficacy of daptomycin on MRSA biofilms could be increased by synergetic interaction with the antimiocrobial cationic peptide nisin (42).

**Table 1:** **Basic characteristics of the MRSA active antibiotics vancomycin, linezolid, and daptomycin**.

	Vancomycin	Linezolid	Daptomycin
Chemistry	Glycopeptide	Oxazolidinone	Cyclic lipopeptide
Activity	Bactericide	Bactericide	Bactericide
Target	Cell wall	Protein synthesis	Cell membrane
Application	i.v.	i.v. and per oral	i.v.
Daily dosage^a^	2 × 1,000 mg (or 4 × 500 mg)	2 × 600 mg	1 × 4 mg/kg

*^a^ According to the recommendations of producer; i.v., intravenous*.

Interestingly, *S. aureus* is stimulating the production of inflammatory cytokines (43). Antibiotics may have immunomodulatory properties during an infection. In an experimental *in vitro* study on peripheral blood mononuclear cells, Pichereau et al. found that many different antibiotics tended to reduce the production of cytokines after toxin exposure (43). Some of these drugs were vancomycin, linezolid, daptomycin, clindamycin, and tigecycline. The inhibiting effects of daptomycin at clinical serum peak concentrations (*c*_max_) on the production of interleukin-1β (IL-1β), IL-6, IL-8, interferon-γ, and tumor necrosis factor alpha (TNF-α) were variable. The suppression of cytokine production was by most antibiotics concentration-dependent but not in the case of daptomycin (and vancomycin). Thallinger and colleagues, in an experimental model of human endotoxemia, found no effect of daptomycin on levels of IL-1β, IL-6, and TNF-α probably due to a high affinity of daptomycin to bacterial cytoplasmic membrane and its low potential to penetrate into human cells (44).

A new very interesting aspect of immunomodulation of daptomycin is related to immune enhancement. Vitamin E is an immune enhancer. Salinthone and colleagues found immunomodulatory properties of vitamin E also in human peripheral mononuclear cells due to alteration of cytokine production (IL-2, IL-8, and IL-17) in part by stimulating production of cyclic adenosine monophosphate (cAMP) (45). In an animal model, administration of vitamin E before infecting wounds by MRSA improved the efficacy of daptomycin (46). Furthermore, Pierpaoli et al. found that if animals were treated with vitamin E before the wounds were infected with MRSA, the animals showed significantly increased CD49b+ cells after the application of daptomycin while daptomycin alone did not change leukocyte populations (47).

Unfortunately, there is not enough clinical data available regarding immunomodulation in patients, but clinical studies demonstrated that “clinical failure in daptomycin-treated skin and soft-tissue infections is associated with severity of infection,” e.g., sepsis, intensive care unit stay, and renal insufficiency (27). These findings support the hypothesis that reduced immune responses correlate directly with MRSA vulnerability. The result is a vicious cycle compromising the immune response ability of the patient and in turn leading to a further deterioration of clinical condition. However, it is very interesting to think about possibilities to break this vicious cycle. Of course, antibiotics “kill” bacteria directly but could, for example, the application of vitamin E support the efficacy of daptomycin in humans? And if yes, when should the application of vitamin E, take place, simultaneously with daptomycin or before the antibiotic treatment? In the previously mentioned experimental studies, animals were treated with vitamin E before the wounds were infected by MRSA. Should we therefore perhaps pre-treat all patients with vitamin E or at least patients at risk of MRSA colonization before any surgery? At the moment, we do not have answers to these questions but these questions may stimulate further clinical research on this topic. The results from experimental studies are very encouraging.

## Concluding Remarks

Infections after open-heart surgery are complications seriously endangering survival. Wounds infected by MRSA are extremely difficult to manage due to their resistance to many antibiotics. Vancomycin is the first-choice antibiotic but only available intravenously. Linezolid is also available per oral and has comparable effects. Daptomycin has good tissue penetration and is more effective in biofilm. The latter effect may be important in treatment after open-heart surgery due to surgical implants. Although clinical evidence is limited, it suggests that daptomycin causes immunomodulation suppressing dose-independent cytokine production after stimulation by MRSA. In experimental studies, immune enhancers (e.g., vitamin E) increased efficacy of daptomycin.

## Conflict of Interest Statement

The author declares that the research was conducted in the absence of any commercial or financial relationships that could be construed as a potential conflict of interest.
